# Patellar Tendon Structural Difference Occurs in Female and Male Professional Basketball Players: 8 Months Follow-Up

**DOI:** 10.3390/jfmk10040420

**Published:** 2025-10-24

**Authors:** Silvia Ortega-Cebrián, Caritat Bagur-Calafat, Cristina Adillón, Silvia Treviño, Carles Martin, Javier Ruiz, David Urbano, Gil Rodas

**Affiliations:** 1 Physiotherapy Department, Facultat Fisioteràpia, Universitat Internacional de Catalunya, 08017 Barcelona, Spain; 2 Department of Medicine and Surgery, Faculty of Medicine and Health Sciences, Institut d’Investigació Sanitària Pere Virgili, Universitat Rovira i Virgili, 43204 Reus, Spain; 3 Health Department, Catalan Basketball Federation, 08018 Barcelona, Spain; 4 Futbol Club Barcelona, Medical Department, Ciutat Esportiva Joan Gamper, Avinguda, Once Setembre, 08970 Barcelona, Spain; 5 Sports and Exercise Medicine Unit, Hospital Clinic and Sant Joan de Déu, 08006 Barcelona, Spain

**Keywords:** tendinopathy, basketball, imaging, sport injury, women

## Abstract

**Background:** Tendon structure is related to the magnitude of load and its management, rather than directly predicting pain incidence. Although pain symptoms frequently persist until they severely impact performance, function, and tendon structure, professional basketball players often manage patellar tendon pain alongside high training and competition loads. The aim of this study was to investigate patellar tendon structural adaptations over 8 months of training and competition in professional female and male basketball players. **Methods:** The primary outcome of this study was defined as the change in the percentage of echo-type II structure from baseline to 8 months. A prospective cohort study was conducted where 43 professional basketball players (20 male, 23 female) were followed during 8 months of training and competition. A bilateral patellar tendon ultrasound tissue characterization (UTC) scan was conducted, and jumping leg (jumping/non-jumping), presence of pain (yes/no), and exposure time (hours) were recorded at baseline, and at 4 and 8 months in the competitive season. **Results:** The mid-tendon exhibited negative adaptations (decreased alignment), represented by echo-type II, after 8 months of competition (left; *p* = 0.001; ES = 2.16; right; *p* = 0.001; ES = −1.35). Positive adaptations (increased alignment], were observed in echo-types III (*p* = 0.004; ES = −1.04) and IV (*p* = 0.001; ES = −0.15), after 4 and 8 months. Tendon structure showed differences between female and male professional basketball players throughout the 8 months (echo-type II; *p* = 0.00; ES = 0.34). **Conclusions:** The study demonstrated that the tendon structure undergoes significant adaptations, supporting the concept that the patellar tendon adapts to compensate for areas of structure disorganization. Female professional basketball players appeared to maintain a more organized tendon structure than their male counterparts. Jumping leg and presence of pain did not show significant differences in tendon structure over the study period. This research has significant implications for elite sport, as a better understanding of tendon load capacity throughout a competitive season is needed.

## 1. Introduction

Patellar tendinopathy is a prevalent cause of anterior knee pain and functional impairment in sports. It is particularly common in jumping and high-intensity sports that involve high tensile loading of the patellar tendon, such as acceleration and changes in direction, often due to chronic overload [1,2,3]. Historically, the patellar tendon pain was primarily linked to jumping activities, known as “jumper’s knee” [4,5].

This condition, defined as pain at the proximal pole of the patella, in athletes involved in repetitive jumping, such as volleyball and basketball players, remains closely associated with patellar tendinopathy [5]. Diagnosis is challenging due to the lack of a single definitive diagnostic test, comorbid pathology, and limitations in imaging techniques, which hinder the ability to reliably distinguish between symptomatic and asymptomatic structural changes [6].

In high-performance sports, particularly in basketball culture, a certain level of patellar tendon pain has become normalized [7]. Basketball is a high-intensity, intermittent sport, characterized by running, sprinting, continuous changes in direction, dribbling, and jumping, which are mechanisms related to patellar tendinopathy [8]. Hutchison et al. (2019) reported that approximately one in three male collegiate basketball players exhibits patellar tendinopathy or patellar structural abnormalities [8]. Epidemiological studies indicate that while only 5% of tendinopathies necessitate time-loss from training and competition, a significant majority (64.4% to 80%) of affected athletes continue to train and play [7,9]. In an 8-year follow-up study, patellar tendinopathy was the most frequent tendinopathy in professional basketball players, with an incidence of 22.7% [9]. Regarding sex differences, a higher incidence has been reported in male players than in females, although no difference in time-loss from training or competition has been noted [7]. Professional basketball showed different load profiles between sex; load profiles for female basketball players during games include approximately 1.0 jumps per minute [/min], 1.7 to 7.3 runs/min, 1.7 to 2.7 sprints/min, 0.84 dribbles/min, and movement changes every 2–3 s [10,11]. In contrast, male players spend an estimated 3.3–4.9% of game time sprinting, 33.6% running, 10.6% dribbling, and 2.3% jumping [12,13]. Correlations between sex, load, and tendon structure remains unknown. For both sexes, acute excessive loading and long-term tendon loading, coupled with a failed healing process, are considered key risk factors for developing patellar tendon pain and structural changes, including tendon thickening, fibrillar disorganization, and neovascularization [1,14,15], which have been observed in professional basketball players, but never reported as sex differences [9,16]. It is hypothesized that tendon thickening may represent a compensatory attempt to align a greater number of parallel collagen bundles to offset disorganized ones [17,18]. However, whether this compensatory mechanism extends to mechanical properties remains unclear [19].

Regarding tendon structure, the degree of disorganized collagen can be objectively measured using Ultrasound Tissue Characterization (UTC). UTC is a relatively new technology that classifies fibrillar organization into four echo-types, ranging from parallel, highly organized collagen bundles (echo-type I) to completely disorganized collagen bundles (echo-type IV) [20]. While UTC is not a diagnostic device, it provides objective, quantitative information about changes in internal tendon structure [21]. UTC has been used to monitor tendon adaptation, finding inconsistent effects of training load on changes in the tendon structure [19]. For instance, no immediate effect on tendon adaptations was observed in the Achilles and patellar tendons following an acute training load [22]. However, changes in tendon structure after pre-season and mid-term training have been demonstrated in team sports athletes [23,24,25]. Further tendon adaptations have been found in the patellar tendon; a decrease in the most organized echo-type I and an increase in echo-type II have been noted in the dominant leg of volleyball players after 7 weeks of monitoring training and game load [23]. Similarly, structural adaptations have been observed in the patellar tendon of handball players after pre-season training [24]. Steinberg et al. (2022) reported an improved tendon structure after 14 weeks of training in healthy soldiers, occurring 40% in the Achilles tendons and in less than 10% of the patellar tendons [25]. In addition, other studies found no structural differences, analyzed with UTC, despite observing tendon thickening in professional and amateur basketball players with US [16]. 

The influence of cumulative load on the patellar tendon structure and tendinopathy in professional basketball players is still poorly understood. Investigations have primarily focused on male participants, and a lack of sex-differentiated data persists in the scientific literature. While growing evidence supports monitoring load to promote positive tendon adaptations, more research is needed to study the effects of a season on structural tendon changes in elite professional female and male basketball players, to elucidate the clinical relevance of this response. This research has significant implications for elite sport, as a better understanding of tendon load capacity is crucial for optimizing training loads, enhancing performance, and reducing the risk of tendinopathy. These insights can help physiotherapists and strength and conditioning coaches make evidence-based decisions about how season-long training and competition loads affect tendon structure and athlete longevity. We hypothesize that the tendon adapts to load along a competitive season, and that males suffer from further tendon structural changes than females in professional basketball players. Therefore, the primary aim of this study was to investigate patellar tendon structural adaptations across a full competitive season in professional basketball players. The secondary aims were to identify differences in tendon structure based on sex, dominant jumping leg, and the presence of pain at baseline, and at 4 months and 8 months of competition. This is the first original study to provide detailed information about tendon adaptation differences between professional female and male basketball players.

## 2. Materials and Methods

### 2.1. Study Design

Four professional basketball teams, comprising both female and male players, competing in national and international competitions, participated in the study. A total of 34 female and 37 male professional basketball players, aged between 18 and 30 years, participated in this prospective cohort study. Reporting follows Strengthening The Reporting of Observational Studies in Epidemiology (STROBE) Guidelines [26].

### 2.2. Inclusion Exclusion Criteria

All male participants were recruited between September 2019 and April 2021, and female participants between September 2022 and April 2023. Players who could not complete three UTC scans within a season, long-term-injured players, and players transferred to other teams were excluded from the study. All participants provided signed informed consent. The study was performed in accordance with the ethical standards of the Helsinki Declaration, and ethical approval was obtained by the Ethics Committee for Clinical Research of the Catalan Sports Council (015-CEICGC-2022).

### 2.3. Procedure

To minimize disturbance to the professional players’ highly demanding schedules and reduce data collection time for UTC scans, the following practical decisions were made: (a) scanning was performed either before or after training, a decision supported by evidence that echo-type changes are not immediately apparent following high-exercise demands [22,26]; (b) data collection took place in the team’s physiotherapy room under the supervision of the team’s physiotherapist; (c) data concerning demographic information, hours of exposure, and presence of pain were collected by the team’s physiotherapist and sport conditioning coach; and (d) the follow-up time was set at 8 months from baseline to avoid data collection during the final, critical competitions of the season, ensuring the viability of the study.

Demographic data (height, weight, and player position) and jumping leg dominance data were provided by the team’s physiotherapist at baseline only. Data concerning presence of pain and total time exposure in training and competition, and UTC scans were recorded at baseline, 4 months, and 8 months. Presence of pain and training hours were provided by the team’s physiotherapist and physical coach, respectively. Training exposure (hours) was documented using objective team data, including training attendance records, staff logs, and Global Positioning System (GPS) metrics, all managed directly by the team staff. Match exposure (minutes) was obtained from the official website of the Spanish Basketball Federation (www.feb.es) by the PI. Total exposure time (hours) was calculated by summing the total documented training hours and the minutes accumulated during official matches, converted to a single hourly metric.

**Figure 1:**
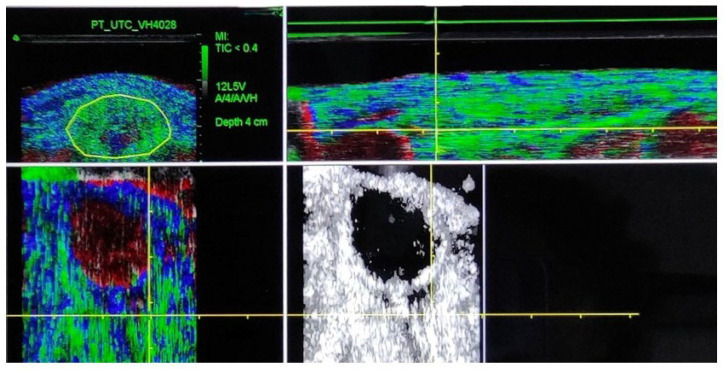
Ultrasound tissue characterization of the patellar tendon. Echo type classification; green = echo type I, blue= echo type II, red = echo type III and black = echo type IV: Right upper imagen (area of interest) = transversal view; left upper image = longitudinal view; right lower image = axial view; left lower image = axial view without UTC software classification.

Bilateral UTC scans of the patellar tendon were conducted at the following three times during one season: at the start of the season (baseline), four months, and eight months after the baseline scan. All tests were performed by a single, experienced tester (SOC) to minimize potential inter-tester bias [27]. UTC scans were performed using B-mode ultrasound with a linear transducer of 7–10 MHz (SmartProbe 10L5; Terason 2000, Teratech, Burlington, MA, USA). An ultrasound probe (SmartProbe 12L5-V, Terason 2000+; Teratech) was fixed to a tracking device (UTC Tracker, UTC Imaging) that automatically moved the transducer on the perpendicular axis of the tendon, recording cross-sectional images at 0.2 mm intervals [27]. The consistency of the intensity and distribution of gray images was calculated over a 4.8 mm window, using UTC algorithms to define the echo-patterns. Four echo-types were identified based on the consistency of gray images, with echo-types I and II representing the most continuous/aligned tendon structure, and echo-types III and IV representing the more unstructured/disorganized tendon fibers. Echo-types are reported as a percentage of the total region of interest (ROI) volume. A window size of 17 was used for imaging analysis. The ROI was located around the tendon in the transverse view, with contours drawn at the proximal tendon (20% of tendon length) and the mid-tendon (50% of tendon length) [16,24]. Total tendon length was measured from the inferior angle of the patella to the most proximal part of the tibial tuberosity [16,21,24]. Tendon contours were marked by an experienced investigator (SB) to increase the reliability of UTC outcomes [28]. Echo-types were quantified automatically by the UTC software (UTC 2010) [21] (Figure 1).

### 2.4. Statistical Analysis

Patellar tendon structure differences for the proximal and mid-tendon were calculated as the change from baseline (i.e., measurement at 4 or 8 months minus baseline) for echo-types I, II, III, and IV, respectively, represented by the size of the changes in percentage. The primary outcome of this study was defined as the change in the percentage in echo-type II structure from baseline to 8 months. Data are presented using the median and interquartile range (IQR) for each echo-type at the three time points (baseline, 4 months, and 8 months). For the change scores, negative values for echo-types I and II indicate a loss of continuity of tendon structure and poorer tendon adaptations (more disorganization), while negative values for echo-types III and IV indicate a lower percentage of disorganized fibers, suggesting a better tendon structure and healthier tendon adaptation changes (more organization).

The Shapiro–Wilk test indicated that the data did not follow a normal distribution; thus, non-parametric tests were used for descriptive statistics. Due to the inherent constraints of the echo-type data (echo-type I + II + III + IV = 100%), we applied the Isometric Log-Ratio (ILR) transformation to the four echo-type percentages, yielding four unconstrained, independent variables. We conducted a Multivariate Mixed Linear Model (MMLM) to evaluate the effect of time (baseline, 4, and 8 months) and the influence of fixed factors (sex, jumping leg, presence of pain, and season/cohort) on the overall tendon composition, represented by the four ILR-transformed variables. Additionally, 95% confidence intervals and values for each parameter were considered. All model assumptions, including multivariate normality of residuals, were verified. Significant effects were subsequently mapped back into the original compositional space for clinical interpretation. The magnitude of the difference between groups was quantified using the standardized mean difference (Cohen’s d). The potential interactions between factors were also evaluated. To assess whether the observed differences were clinically meaningful, the Minimal Detectable Change (MDC) values for each echo-type were calculated. The standard error of measurement (SEM) was calculated as SEM (SEM = Standard deviation × √(1 − ICC)), and MDC was calculated as MDC (MDC = 1.96 × SEM × √2). Intraclass correlation coefficient (ICC) values from the authors’ previous studies using the same methods were utilized. High reproducibility and moderate to excellent intra-observer reliability have been reported with UTC (ICC) in trained observers [21,24]. The alpha level for statistical significance was set at 0.05. All statistical calculations were performed using R (version 4.5.0) Statistical Software.

## 3. Results

From the original cohorts of 37 male and 34 female players, 43 participants (20 males and 23 females) were included in the final analysis, after completing all UTC scans within the season. Players were excluded due to poor-quality scans showing artifacts or anisotropic images. To confirm the adequacy of the sample size, a post hoc power analysis was conducted, using a power of 80% and a significance level of 0.05. A total of 19 participants would have been required to demonstrate significant changes in echo-type I (effect size = 3.48).

A summary of demographic data is presented in Table 1. Significant demographic differences were found between cohorts in height, weight, and time of exposure after 4 months. Individual demographic and descriptive data for all echo-types at the proximal and mid-tendon, as well as percentages of the tendon structure difference for each echo-type, are available as Supplementary Materials. 

To interpret the results regarding tendon adaptations, it is important to note the convention for effect size (ES); a negative ES for echo-types I and II signifies a decrease in organized structure (more disorganization), whereas a negative ES for echo-types III and IV signifies a decrease in disorganized structure (more organization). Complete numeric data of percentage structural tendon differences, including Median (IQR), 95% CI, MDC, *p*-value, and effect size, are shown in Table 2.

### 3.1. Tendon Adaptations

The results relating to the primary aim—identifying tendon adaptations along a competitive season—showed no significant adaptations in the tendon structure of the proximal tendon after 4 months. For the mid-tendon, at 4 months, negative adaptations were observed only for echo-type II (left; *p* = 0.004; ES = −0.13).

After 8 months, the proximal tendon showed positive adaptations for echo-type I (left; *p* = 0.005; ES = 1.14) and negative for echo-type II (left; *p* = 0.002; ES = −0.35); no further differences were seen in echo-type III and IV. The increased percentage for echo-type I was seemingly compensated by the decreased percentage for echo-type II. Consequently, overall proximal tendon adaptations after 8 months showed an increased percentage of tendon disorganization occurring in echo-type II.

The most consistent and significant adaptations occurred at the mid-tendon after 8 months for all echo-types, as follows: echo-type I (right; *p* = 0.03; ES = 1.08), echo-type II (left; *p* = 0.001; ES = −2.16; right; *p* = 0.001; ES = −1.35), echo-type III (right; *p* = 0.004; ES = −1.04), and echo-type IV (right; *p* = 0.01; ES = −0.15). The study demonstrates that most of the tendon adaptation occurs in the mid-tendon after 8 months of competition. Greater positive adaptation patterns were seen in echo-types I, III, and IV, despite localized tendon disorganization in echo-type II.

### 3.2. Tendon Structure Differences Between Sexes

The results relating to the secondary aim—tendon structure differences between sexes—were observed throughout the season. At baseline: in the proximal tendon, women presented with less echo-type II (ES = 0.21; *p* = 0.04). In the mid-tendon, differences were seen in echo-type I (ES = −0.16; *p* = 0.02), where women presented a more organized tendon structure than men. At 4 months: at both the proximal and mid-tendon, women presented a higher percentage of echo-types III and IV than men (proximal: ES = 0.19; *p* = 0.00; ES = 0.24; *p* = 0.00; and mid-tendon: ES = 0.23; *p* = 0.00; ES = 0.19; *p* = 0.00). At 8 months: at the proximal tendon, women demonstrated a higher percentage of the most organized echo-type I (ES = −0.25; *p* = 0.001), and lower percentage of echo-type II (ES = 0.11; *p* = 0.05). Echo-type III (ES = 0.57; *p* = 0.00) was higher in women, while echo-type IV was lower (ES = 0.56; *p* = 0.00). At mid-tendon, women showed a lower echo-type II (ES = 0.34; *p* = 0.00), higher echo-type III (ES = 0.35; *p* = 0.00), and lower echo-type IV (ES = 0.28; *p* = 0.00), compared to men.

No correction effects were found for hours of exposure, jumping leg, developed pain, or season/cohort when analyzing sex differences (See Table 3).

### 3.3. Tendon Structure Differences Between Jumping and Non-Jumping

Tendon structure differences between the jumping and non-jumping leg were only seen at the proximal tendon during baseline for echo-type III (ES = −0.30; *p* = 0.01) and IV (ES = −0.26; *p* = 0.01). The negative effect size corresponds to the higher echo-type III and IV in the non-jumping leg, indicating there were more disorganized tendon fibers in the non-jumping leg at the start of the season. These differences were not sustained at 4 or 8 months.

### 3.4. Tendon Structure Differences Between Symptomatic and Asymptomatic

Differences between symptomatic (presence of pain) and asymptomatic tendons were not found at the proximal tendon (20% of tendon length). Symptomatic tendons showed a greater proportion of disorganized tendon structure, with differences seen only at the mid-tendon at baseline for echo-type II (ES = −0.15; *p* = 0.05), and at 4 and 8 months for echo-type I (ES = −0.26; *p* = 0.04, ES = −0.36; *p* = 0.05, respectively).

### 3.5. Tendon Structure Differences Between Season/Cohort

The effect of the cross-seasonal data collection was found to be statistically non-significant, indicating that the timing of data collection did not significantly contribute to the overall variance in tendon structure changes (see Supplementary Data).

Table 4 presents a summary table of the statistically significant differences in patellar tendon structure in echo-types I–IV, the difference between female and male basketball players, jumping and no-jumping leg, symptomatic and asymptomatic, and season and cohort for tendons of the proximal and mid-tendon area at baseline, and at 4 and 8 months of training and competition.

## 4. Discussion

The primary aim of this study was to investigate patellar tendon structural adaptations over 8 months of training and competition in professional female and male basketball players. This is the first study to compare objective patellar tendon structures across a full competitive season and to investigate differences between professional female and male basketball players using UTC. These findings underscore the importance of considering factors such as sex when evaluating tendon adaptations, and suggest potential avenues for targeted interventions.

Structural adaptations within the proximal tendon following a four-month training and competitive phase were not statistically significant. Although non-significant, a detrimental trend in echo-type II, indicative of tissue disorganization, was observed in this proximal region during the initial competitive cycle. In contrast, the mid-tendon exhibited statistically significant alterations in echo-type II, suggesting negative structural adaptations in response to cumulative mechanical loading. Intriguingly, this localized negative change appears to have been potentially counterbalanced by positive changes and effect sizes observed in echo-type I, indicative of superior fibrillar organization. This observed inverse relationship—specifically, a decrease in echo-type II concurrent with an increase in echo-type I—diverges from previously documented patterns in the literature [23,25], which have reported opposing adaptations (negative in echo-type I and positive in echo-type II). These changes may be attributable to the tendon’s inherent capacity for adaptation between the highly organized states represented by echo-types I and II. Tendon tissue has established mechanisms to revert towards a more organized fibrous structure, modulated by factors such as mechanical load tolerance and sufficient recovery periods [18,29]. In contrast, previous research on short-term training periods demonstrated structural changes as a response to pre-season or high-intensity exercise bouts, but it remains unclear whether the tendon consistently adapts towards a more organized or disorganized state [17,22,24,25,26].

After 8 months of competition, structural tendon adaptations at the proximal tendon showed positive adaptations for echo-type I and negative adaptations for echo-type II. Importantly, these structural changes generally remained within the optimal of echo-types I and II, which is considered characteristic of a healthy tendon structure [16]. The MDC and effect size data collectively suggest positive adaptations across all echo-types, compensating for the decreased percentage of echo-type II. The most consistent and significant echo pattern occurred at the mid-tendon after 8 months, confirming the pattern of decreased echo-type II being compensated by the improvement (organization) of all other echo-types. These results confirm that cumulative tendon load during a season appears to increase tendon structural stability, primarily for echo-types I and II. The clinical relevance of our results confirms the dynamic capacity of the mid-tendon structure to reverse from misaligned tendon structure after the long-term effects of load, preserving greater structural integrity in professional basketball players. Further research is warranted to investigate these structural changes throughout the maturity years of professional basketball practice, and their clinical presentation.

Given our results, the proximal tendon showed minimal differences, with the main structural changes occurring at the mid-tendon. While changes in tendon tissue and patellar tendinopathy are commonly found at the origin or insertion of symptomatic or asymptomatic tendons [2], our findings suggest that the structural response to load may occur to a lesser or slower extent at the insertional tendon. Load distribution throughout the tendon, coupled with the long-term effects of chronic load, could explain the greater structural response at the mid-tendon compared to the proximal tendon. Additional research is necessary to fully explore the differential structural response to load across different tendon portions.

Sex Differences in Tendon Structure

When comparing professional female and male basketball players at baseline, female players showed a lower proportion of structural fibers in echo-type II at the proximal tendon and a more aligned echo-type I at the mid-tendon. After 4 months of competition, female players showed a greater proportion of the more disorganized echo-types III and IV than male players, at both the proximal and mid-tendon. However, after 8 months of competition, female players generally maintained a more organized structural fiber profile across all echo-types than males at the proximal tendon. At the mid-tendon, female players demonstrated greater tendon integrity for echo-types II, III, and IV compared to males.

This sex difference may be explained by disparities in sport demands and cumulative training loads between professional female and male basketball players. Ark et al. (2015) reported differences in echo-type II between male and female adolescent volleyball players after a 5-day tournament [22]. Furthermore, adolescent female elite ballet dancers have shown greater thickness in the proximal patellar tendon, arguing for the importance of load exposure during growth ages [17]. We hypothesize that the intensity of training and exposure during adolescence may be less pronounced in female basketball players than in males, which could predispose adult female professional athletes to better long-term tendon integrity. Authors are unable to draw any conclusions regarding the relationship between our findings and hormonal changes related to tendon stiffness. Firstly, because the theoretical link between these specific hormonal factors and patellar tendon stiffness has yet to be definitively established in the current literature, and, secondly, because tendon stiffness does not imply tendon structural changes found using UTC, which was the main topic of the study. It is also important to note that the cross-seasonal timing of our data collection could not be accurately mapped to any specific moment of the menstrual cycle for our female participants, which prevents us from making any conclusive links between our findings and potential hormonal fluctuations.

Our study is unique in demonstrating a significant difference in structural tendon response to long-term load between sexes in professional basketball players.

Other Factors: Jumping Leg and Pain

Tendon structure differences between the jumping and non-jumping leg showed greater disorganized tendon fibers (echo-types III and IV) in the non-jumping leg only at baseline. These results contradict Rabello et al.’s (2019) study, where volleyball players exhibited decreased echo-types I and II in the jumping leg, with no changes in the non-jumping leg [23]. Considering the diverse sport demands of basketball (accelerations, decelerations, sprint, and changes in direction) compared to the predominance of jumping in volleyball [19], specific sport demands beyond just jumping activity may influence the structural difference between the jumping and non-jumping legs. We believe that a clearer distinction is needed between the concepts of a dominant leg and a jumping leg to more accurately assess the risk factors for patellar tendinopathy.

Differences in tendon structural changes between symptomatic and asymptomatic tendons were minimal, found only in the proximal tendon for echo-types I and II after 4 and 8 months of competition, and at baseline, respectively. Our results support the existing evidence that there is limited association between structural changes in the tendon and the presence or absence of pain [30,31,32,33]. Differences in proximal tendon structure and pain presence at baseline could be related to pre-season training, as seen in handball players or after high-intensity bouts of exercise [23,24]. In our study, 5 male players (25%) and 4 female players (17.3%) reported pain, with the highest prevalence at baseline, and none required time off from training or competition. Due to the small number of athletes who developed pain, we cannot definitively conclude that pain presence is related to training overload, competition, or structural changes, as has been suggested in previous studies involving volleyball and basketball players [34,35]. Our results support the continuum model of tendinopathy, emphasizing [2] that changes in tendon structure are related to the magnitude of load and its management, rather than directly predicting pain incidence.

A limitation of the present study is its cross-seasonal data collection. Although a ‘season/cohort’ variable was included as a fixed effect in the MMLM to account for these timing differences, we cannot fully exclude the residual influence of environmental factors (e.g., matches and changes in overall league scheduling) or unmeasured factors associated with these different collection periods. Therefore, while our primary findings on sex differences are robust to this factor, the interpretation of certain time-dependent effects should be considered within this context. Authors chose to finalize data collection before national and European final competitions, as team managers restricted measurements to avoid player disturbance before training. Differences in tendon length for UTC analysis between studies could affect the comparison of results, although all methods used have been previously reported. The low incidence of pain limited the ability to draw robust conclusions regarding the relationship between pain and structural changes.

## 5. Conclusions

The current study supports the concept that the tendon adapts to compensate for areas of disorganization, primarily in the mid-tendon, after 8 months of competition. Female professional basketball players appear to maintain a more organized tendon structure than males. Neither the dominant jumping leg nor the presence of pain demonstrated significant longitudinal changes in tendon structure at 4 and 8 months of training and competition.

## Figures and Tables

**Table 1:** Participants’ demographics.

Gender (n)	Female (23)	Male (20)
Height (Kg) (mean ± SD)	180.91 ± 9.56	199.76 ± 7.98 *
Weight (cm) (mean ± SD)	74.7 ± 6.82	89.22 ± 7.11 *
Position (n/%)		
Shooting Guard	5 (21.73%)	4 (20%)
Pivot Wing	5 (21.73%)	6 (30%)
Small Forwards	6 (26.08%)	5 (25%)
Point Guard	4 (17.39%)	4 (20%)
Center	3 (13.04%)	1 (5%)
Jumping leg (n/%)		
Right	19 (82.6%)	16 (80%)
Left	3 (13.04%)	4 (20%)
Patellar Tendon Pain (n/%)		
Right (Baseline)	4 (17.3%)	5 (25%)
Right (1st Half)	2 (8.6%)	1 (5%)
Right (All Season)	3 (13.04%)	3 (15%)
Left (Baseline)	2 (8.6%)	2 (10%)
Left (1st Half)	1 (4.3%)	2 (10%)
Left (All Season)	2 (8.6%)	3 (15%)
Exposition (h)		
1st Half	77.47 (35.98)	94.33 (27.34) *
All Season	221.54 (48.12)	258.33 (41.27)

Data presented in mean ± standard deviation (SD); cm (centrimeters); Kg (Kilograms); h (hours): * = *p* < 0.005.

**Table 2:** Proximal and mid-tendon structural difference after 4 and 8 months follow-up echo-type I-IV and each tendon).

Time of Season	Tendon Length (%)	Echo-Type	Side	Median (IQR)	95% CI		*p*-Value	Cohen’s d	SEM	MDC
					Sup Lim	Inf Lim				
4 months	Prox Tendon	Type I	L	9.15 (14.34)	10.29	−3.49	0.43	0.65	0.25	2.61
			R	7.97 (17.01)	0.26	−0.86	0.63	0.52	0.16	3.10
		Type II	L	−5.04 (16.82)	0.36	−0.13	0.39	−0.86	0.23	3.90
			R	−1.1 (4.3)	0.43	0.86	0.24	−0.69	0.82	0.78
		Type III	L	0 (0.86)	0.42	0.24	0.39	0.44	0.92	0.82
			R	0 (0.41)	0.66	−0.66	0.76	−0.38	0.69	0.76
		Type IV	L	0 (0.87)	0.41	0.98	0.81	−0.42	0.57	0.16
			R	0 (0.61)	0.93	−0.24	0.89	0.58	0.29	0.04
	Mid-Tendon	Type I	L	6.41 (17.62)	0.10	−0.29	0.70	1.00	0.63	0.58
			R	9.93 (26.84)	0.87	−0.34	0.92	0.77	0.55	4.60
		Type II	L	−8.88 (15.64)	0.37	−0.04	0.04 *	−0.13	0.02	2.15
			R	−4.15 (13.86)	0.90	−0.91	0.18	−0.11	0.14	0.29
		Type III	L	0 (0.13)	0.76	0.70	0.76	0.37	0.72	0.64
			R	0 (0.1)	0.45	0.82	0.55	−0.34	0.80	0.61
		Type IV	L	0 (0.69)	0.40	−0.48	0.55	0.26	0.99	0.86
			R	0 (0.52)	0.37	0.97	0.16	−0.86	0.54	1.82
8 months	Prox Tendon	Type I	L	5.05 (28.93)	0.61	−0.94	0.05 *	1.14	0.77	2.54
			R	8.31 (13.97)	0.88	−0.76	0.88	−0.18	0.75	1.79
		Type II	L	−5.31 (25.64)	0.36	0.93	0.02 *	−0.35	0.92	2.31
			R	−1.53 (12.3)	0.15	0.08	0.62	0.33	0.67	0.48
		Type III	L	0 (0.07)	0.16	−0.10	0.06	0.71	0.09	0.23
			R	0 (0.26)	0.11	0.64	0.28	−0.17	0.77	0.08
		Type IV	L	0 (0.12)	0.63	0.06	0.89	−0.13	0.54	0.06
			R	0 (0.69)	0.71	−0.28	0.23	0.83	0.36	1.36
	Mid-Tendon	Type I	L	2.83 (25.93)	0.91	−0.37	0.09	0.60	0.77	1.75
			R	6.95 (23.01)	0.09	0.21	0.03 *	−1.08	0.59	2.70
		Type II	L	−1.51 (17.78)	0.38	−0.84	0.01 *	2.16	0.95	1.24
			R	−4.61 (19.42)	0.64	0.36	0.01 *	−1.35	0.65	0.31
		Type III	L	0 (0.31)	0.17	−0.01	0.64	0.31	0.36	0.59
			R	0 (0.48)	0.34	0.17	0.04 *	−1.04	0.04	0.15
		Type IV	L	0 (0.57)	0.31	−0.40	0.91	0.16	0.81	0.27
			R	0 (0.87)	0.42	−0.16	0.01 *	−0.15	0.02	0.05

L = Left tendon; R = Right tendon; (IQR) = Inter quarter range; CI = Coefficient interval; Sup Lim = Superior limit; Inf Lim = Inferior Limit; SEM = Small Error of Measurement; MCD = Minimal Detectable Change; and * = *p* value < 0.05.

**Table 3:** Tendon structure differences between female and male players.

			Male		Female		
		Echo-Type	Median (IQR)	95% CI (Sup to Inf)	Median (IQR)	95% CI (Sup to Inf)	Effect Size
Baseline	Proximal Tendon	Type I	−2.7 (22.2)	(7.07 to −25.47)	−4.4 (37.7)	(12.44 to 0.01)	−0.17
		Type II	0.4 (24.7)	(20.32 to −2.32)	−3.6 (22.2)	(2.933 to −4.4)	0.21 *
		Type III	0 (0.5)	(2.77 to −0.37)	0.04 (2.24)	(1.797 to −0.2)	0.01
		Type IV	0 (0.3)	(0.5 to 0.02)	0.2 (0.8)	(0.934 to 0.07)	−0.04
	Mid-Tendon	Type I	−3.5 (27.1)	(2.38 to −22.72)	4.6 (24.3)	(13.92 to −0.6)	−0.16 *
		Type II	3.4 (27.9)	(22.25 to −2.38)	−0.24 (23.74)	(4.141 to −6.3)	0.11
		Type III	0 (0.05)	(0.2 to −0.02)	0.2 (2.6)	(1.124 to −0.2)	−0.04
		Type IV	0 (0)	(0.04 to 0)	0.04 (0.7)	(0.346 to 0)	−0.07
4 months	Proximal Tendon	Type I	−3.45 (34.2)	(23.45 to −22.34)	20 (24.8)	(13.33 to 1.34)	−0.1
		Type II	2.25 (26)	(5.34 to −3.48)	−8.74 (22.84)	(3.929 to −13)	0.02
		Type III	0.2 (2.4)	(2.77 to −0.73)	−2 (2.4)	(−0.33 to −2.3)	0.19 *
		Type IV	0 (0.3)	(0.8 to −0.08)	−0.34 (0.4)	(−0.27 to −1)	0.24 *
	Mid-Tendon	Type I	−0.4 (35.05)	(22.27 to −22.83)	22.74 (22.97)	(16.77 to 4.66)	−0.16
		Type II	0.05 (20.36)	(4.5 to −22.22)	−22.34 (8.2)	(−4.12 to −14)	0.23
		Type III	0.05 (0.75)	(0.75 to 0.22)	−0.44 (2.2)	(−0.32 to −1.7)	0.23 *
		Type IV	0 (0.3)	(0.28 to 0.02)	−0.2 (0.6)	(−0.14 to −0.7)	0.19 *
8 months	Proximal Tendon	Type I	−2.4 (20.5)	(4.28 to −22.32)	22.8 (23.4)	(20.94 to 6.79)	−0.25 *
		Type II	2.4 (23.25)	(7.58 to −2.72)	−9.3 (22.4)	(1.133 to −14)	0.11 *
		Type III	0.3 (2.7)	(2.24 to 0.37)	−0.74 (2.2)	(−0.33 to −0.9)	0.57 *
		Type IV	0.2 (2.2)	(2.07 to 0.27)	−0.04 (0.3)	(−0.03 to −0.2)	0.56 *
	Mid-Tendon	Type I	−5.4 (25.75)	(5.42 to −25.3)	23.4 (24.04)	(23 to 9.69)	−0.28
		Type II	3.2 (29.9)	(7.77 to −4.2)	−20.64 (23.24)	(−4.62 to −16)	0.34 *
		Type III	0.25 (0.95)	(0.73 to 0.27)	−0.4 (0.9)	(−0.37 to −1)	0.35 *
		Type IV	0.05 (0.25)	(0.2 to 0.04)	−0.2 (0.3)	(−0.04 to −0.3)	0.28 *

(IQR) = Inter quarter range; CI = Coefficient interval; Sup Lim = Superior limit; Inf Lim = Inferior Limit; and * = *p* value < 0.05.

**Table 4:** Statistically significant difference in the proximal and mid-patellar tendon structural difference after 4 and 8 months, between male and female, between jumping and non-jumping leg, between symptomatic and asymptomatic, and between season and cohort.

	PT Structural Difference (4 Months-Baseline and 8 Months-Baseline)		PT Structural Differences Between Male and Female		PT Structure Differences Between Jumping and Non-Jumping Leg		PT Structural Differences Between Symptomatic and Asymptomatic		PT Structural Differences Between Season/Cohort	
	Proximal Tendon	Mid-Tendon	Proximal Tendon	Mid-Tendon	Proximal Tendon	Mid-Tendon	Proximal Tendon	Mid-Tendon	Proximal Tendon	Mid-Tendon
Baseline			Female players showed greater disorganized tendon in echo-type II	Female players showed more aligned structural fibers in echo-type I	No differences	No differences	No different	Symptomatic tendons showed higher disorganized of echo-type II	No different	No different
4 months	No different	Left tendon: different in echo-type II	Female players show fewer disorganized echo-type IV and greater organization of echo-type III than male	Female players show greater organization of echo-type III and fewer disorganized tendon of echo-type IV than male	Greater disorganized echo-type III and IV at the non-jumping leg	No different	No different	Symptomatic players showed higher disorganized fiber of echo-type I	No different	No different
8 months	Left tendon: disorganized echo-type II and increased echo-type I	Disorganized echo-type II	Female players showed more aligned echo-type I, III and IV than male	Female players show fewer disorganized tendon of echo-type II and IV and greater organization of echo-type III than males	Greater disorganized echo-type III and IV at the non-jumping leg	No different	No different	Symptomatic players showed higher disorganized fiber of echo-type I	No different	No different

## Data Availability

The raw data supporting the conclusions of this article will be made available by the authors on request.

## Supplementary Materials

The following supporting information can be downloaded at: https://www.mdpi.com/article/10.3390/jfmk10040420/s1. Table S1: MMLM with effect baseline, 4, and 8 months and fixed factors (sex, jumping leg, presence of pain, and season/cohort); Table S2: Ultrasound Tissue Characterization (UTC) descriptive data of echo-type (I-IV) at the proximal and mid-tendon at baseline, 4 and 8 months of training and competition professional female and male basketball players.
