# Supplementary material for: Patellar Tendon Structural Difference Occurs in Female and Male Professional Basketball Players: 8 Months Follow-Up
Source: J Funct Morphol Kinesiol. 2025 Oct 24;10(4):420. doi: 10.3390/jfmk10040420 (PMC12641865; doi:10.3390/jfmk10040420)
Supplement: Supplementary file 1 [file jfmk-10-00420-s001.zip › jfmk-3937586-supplementary.pdf]

Table S1: MMLM with effect baseline, 4, and 8 months and fixed factors (sex, jumping leg, presence of pain, and season/cohort)

Difference of % of Echo type (MMLM)

			Male		Female				Jumping Leg		No Jumping Leg				Symptomatic		Asym				2019/ Male		2022/ Female			
			Median (IQR)	95% CI (Sup to In	Median (IQR)	95% CI (Sup to In	Effect Size	p-Value	Median (IQR)	95% CI (Sup to In	Median (IQR)	95% CI (Sup to In	Effect Size	p-Value	Median (IQR)	95% CI (Sup to In	Median (IQR)	95% CI (Sup to In	Effect Size	p-Value	Median (IQR)	95% CI (Sup to In	Median (IQR)	95% CI (Sup to In	Effect Size	p-Value
Type I	Proximal Tendon	Baseline	-2,7 (22,2)	(7,07 to -25,47)	-4,4 (37,7)	(12,44 to 0,01)	-0,17	0,02	6,95 (21,12)	(-5,02 to 10,74)	0,7 (21,65)	(-6,12 to 8,45)	-0,07	0,74	21,29 (10,6)	(4,62 to 1,14)	8,043 (2,39)	(3,11 to -5,52)	-0,17	0,22	20,18(21,29)	(10,14 to 4,94)	10,20(3,69)	(3,5 to -1,81)	0,81	0,11
Type I	Proximal Tendon	4 months	-3,45 (34,2)	(23,45 to -22,34)	20 (24,8)	(13,33 to 1,34)	-0,1	0,41	4,5 (26,2)	(-4,85 to 12)	9,7 (31,1)	(-4,46 to 11,98)	0,01	0,74	29,61 (21,7)	(18,25 to -4,15)	42,6 (14,9)	(29,27 to -11,5)	0,15	0,87	25,77(29,61)	(17,13 to 18,36)	24,90(14,25)	(26,3 to -2,46)	0,46	0,85
Type I	Proximal Tendon	8 months	-2,4 (20,5)	(4,28 to -22,32)	22,8 (23,4)	(20,94 to 6,79)	-0,25 *	0,00	5,3 (13,3)	(0 to 13,14)	5,59 (24,6)	(-4,1 to 14,97)	-0,05	0,46	10,74 (15,9)	(-9,81 to 10,74)	22,77 (27,8)	(5,5 to -5,97)	0,33	0,26	23,24(10,74)	(19,34 to -12,5)	13,33(5,88)	(17,6 to -12,47)	0,47	0,46
Type I	Mid Tendon	Baseline	-3,5 (27,1)	(2,38 to -22,72)	4,6 (24,3)	(13,92 to -0,6)	-0,16 *	0,02	-0,14 (22,4)	(-7,07 to 7,61)	-0,3 (24,5)	(-3,44 to 10,47)	0,14	0,93	6,977 (-9,9)	(5,405 to -16,5)	5,497 (-2,4)	(2,904 to -7,15)	0,06	0,46	51,54(6,977)	(6,75 to 0,38)	32,99(16,73)	(16,9 to -1,76)	0,76	0,39
Type I	Mid Tendon	4 months	-0,4 (35,05)	(22,27 to -22,83)	22,74 (22,97)	(16,77 to 4,66)	-0,16	0,15	7,4 (20,3)	(0,03 to 15,29)	0,59 (20)	(-7,77 to 10,22)	-0,25	0,08	75,24 (-6,9)	(65,66 to -46,2)	13,83 (0,1)	(16,81 to -17,2)	-0,26 *	0,04	36,56(75,24)	(4,82 to 2,12)	19,18(14,19)	(61,10 to -2,61)	0,61	0,80
Type I	Mid Tendon	8 months	-5,4 (25,75)	(5,42 to -25,3)	23,4 (24,04)	(23 to 9,69)	-0,28	0,00	11,2 (22,8)	(2,14 to 14,88)	7,6 (34)	(-5,43 to 17,66)	-0,08	0,44	9,38 (15,67)	(-10,9 to 9,381)	5,54 (26,92)	(-9,25 to -24,4)	-0,36 *	0,05	22,19(9,38)	(16,3 to -0,3)	21,51(18,79)	(23,13 to -7,43)	0,43	0,66
Type II	Proximal Tendon	Baseline	0,4 (24,7)	(20,32 to -2,32)	-3,6 (22,2)	(2,933 to -4,4)	0,21 *	0,05	-5,05 (20,22)	(-7,49 to 3,06)	-0,5 (19,7)	(-2,65 to 8,722)	0,29	0,16	2,7 (0)	(0,951 to -1,44)	0,404 (0)	(0,355 to -1,14)	-0,08	0,31	1,15(2,7)	(0,8 to 0,32)	1,59(1,80)	(1,2 to -2,51)	0,51	0,28
Type II	Proximal Tendon	4 months	2,25 (26)	(5,34 to -3,48)	-8,74 (22,84)	(3,929 to -13)	0,02	0,43	-1,1 (24,2)	(-9,51 to 11,01)	-10 (30,1)	(-12,1 to -0,21)	-0,27	0,15	2,03 (0)	(4,887 to -7)	4,06 (0)	(3,957 to -3,26)	0,62	0,04	1,45(2,03)	(0,32 to 2,13)	1,71(1,86)	(3,1 to -1,33)	0,33	0,35
Type II	Proximal Tendon	8 months	2,4 (23,25)	(7,58 to -2,72)	-9,3 (22,4)	(1,133 to -14)	0,11 *	0,05	-4,6 (16,5)	(-8,66 to 7,05)	-3,4 (31,9)	(-8,73 to 2,948)	-0,10	0,75	1,48 (2,325)	(-0,98 to 1,487)	3,69 (2,65)	(0 to -2,42)	0,11	0,50	0,53(1,48)	(3,1 to -1,1)	0,47(1,14)	(0,67 to -1,56)	0,56	0,64
																									0,12	0,59
Type II	Mid Tendon	Baseline	3,4 (27,9)	(22,25 to -2,38)	-0,24 (23,74)	(4,141 to -6,3)	0,11	0,27	-0,29 (21,67)	(-3,1 to 5,89)	0,45 (21,65)	(-3,68 to 7,488)	0,03	0,64	1,46 (0)	(0,383 to -0,2)	0,183 (0)	(0,13 to -0,38)	-0,15 *	0,02	1,26(1,46)	(0,86 to 5,19)	2,36(4,63)	(1,1 to -2,81)	0,14	0,50
Type II	Mid Tendon	4 months	0,05 (20,36)	(4,5 to -22,22)	-22,34 (8,2)	(-4,12 to -14)	0,23	0,06	-7,5 (18,6)	(-13 to -1,94)	-5,4 (18,7)	(-12,3 to -0,58)	0,06	0,49	0,88 (0)	(2,116 to -3,02)	1,646 (0)	(1,687 to -1,47)	0,58	0,40	0,79(0,88)	(0,98 to 16,34)	12,45(5,86)	(1,2 to -2,46)	0,11	0,37
Type II	Mid Tendon	8 months	3,2 (29,9)	(7,77 to -4,2)	-20,64 (23,24)	(-4,62 to -16)	0,34 *	0,00	-10,2 (20,8)	(-11,1 to -0,6)	-6 (30,5)	(-10,8 to 0,967)	0,05	0,42	0,6 (0,525)	(-0,3 to 0,608)	1,96 (1,22)	(-0,05 to -1,16)	0,17	0,34	0,48(0,6)	(0,89 to -25,2)	8,35(13,76)	(1,1 to -0,47)	0,80	0,47
Type III	Proximal Tendon	Baseline	0 (0,5)	(2,77 to -0,37)	0,04 (2,24)	(1,797 to -0,2)	0,01	0,66	0 (0,9)	(0,05 to 2,2)	0 (1,15)	(-0,39 to 0,673)	-0,36	0,12	15,04 (1,1)	(4,941 to -6,49)	9,873 (-0,89)	(3,094 to -3,61)	-0,02	0,53	17,19(15,04)	(1,76 to 5,41)	11,22(4,62)	(11,11 to -9,76)	0,15	0,17
Type III	Proximal Tendon	4 months	0,2 (2.4)	(2,77 to -0,73)	-2 (2,4)	(-0,33 to -2,3)	0,19 *	0,00	0 (1,3)	(-1,3 to 1,09)	0 (0,4)	(-0,68 to 0,538)	0,01	0,79	16,39 (3,9)	(18,36 to -17,5)	54,47 (14,8)	(46,17 to -30,9)	0,59	0,40	19,42(16,39)	(3,97 to 65,66)	2,54(8,62)	(112,4 to -15,61)	0,50	0,31
Type III	Proximal Tendon	8 months	0,3 (2,7)	(2,24 to 0,37)	-0,74 (2,2)	(-0,33 to -0,9)	0,57 *	0,00	0 (0,6)	(-0,19 to 1,51)	0 (0,9)	(-0,59 to 0,449)	-0,33	0,16	27,86 (39,2)	(-12,5 to 27,86)	24,14 (33,35)	(5,2 to -13,4)	-0,05	0,86	24,7(27,86)	(9,48 to -10,9)	1,19(49,63)	(17,4 to -7,43)	0,78	0,24
Type III	Mid Tendon	Baseline	0 (0,05)	(0,2 to -0,02)	0,2 (2,6)	(1,124 to -0,2)	-0,04	0,49	0 (0,27)	(-0,03 to 0,82)	0 (0,375)	(-0,29 to 0,336)	-0,30 *	0,03	14,06 (-1,4)	(5,193 to -8,56)	4 (2,3)	(2,979 to -8,98)	-0,08	0,75	24,93(14,06)	(4,27 to 4,62)	12,93(32,25)	(2,4 to -1,51)	0,24	0,88
Type III	Mid Tendon	4 months	0,05 (0,75)	(0,75 to 0,22)	-0,44 (2,2)	(-0,32 to -1,7)	0,23 *	0,00	0 (0,5)	(-0,43 to 0,23)	0 (0,3)	(-0,47 to 0,294)	0,01	0,29	16,24 (-3,9)	(16,34 to -13,9)	9,743 (-12,9)	(22,95 to -32,7)	-0,77	0,89	16,70(16,24)	(5,81 to 18,25)	1,6(4,88)	(2,29 to -1,33)	0,90	0,19
Type III	Mid Tendon	8 months	0,25 (0,95)	(0,73 to 0,27)	-0,4 (0,9)	(-0,37 to -1)	0,35 *	0,00	0 (0,7)	(-0,41 to 0,25)	0 (0,5)	(-0,62 to 0,134)	-0,15	0,17	12,99 (38,8)	(-25,2 to 12,99)	2,035 (24,9)	(-12,2 to -23,5)	-0,11	0,74	9,52(12,99)	(4,21 to -9,81)	4,36(5,91)	(-7,18 to -2,56)	0,88	0,24
Type IV	Proximal Tendon	Baseline	0 (0,3)	(0,5 to 0,02)	0,2 (0,8)	(0,934 to 0,07)	-0,04	0,29	0 (0,47)	(0,11 to 0,84)	0 (0,3)	(0 to 0,429)	-0,27	0,17	1,116 (0)	(0,318 to -0,27)	0,193 (0)	(0,321 to -1,2)	-0,23	0,07	1,82(1,116)	(1,42 to 0,95)	1,35(1,19)	(0 to -0,12)	0,24	0,61
Type IV	Proximal Tendon	4 months	0 (0,3)	(0,8 to -0,08)	-0,34 (0,4)	(-0,27 to -1)	0,24 *	0,00	0 (0,3)	(-0,41 to 0,49)	0 (0,2)	(-0,38 to 0,303)	-0,06	0,79	1,616 (0)	(2,134 to -2,33)	1,365 (2,65)	(1,693 to -1,76)	0,08	0,71	1,4(1,616)	(2,66 to 4,89)	1,51(1,79)	(0 to -0,2)	0,76	0,43
Type IV	Proximal Tendon	8 months	0,2 (2,2)	(2,07 to 0,27)	-0,04 (0,3)	(-0,03 to -0,2)	0,56 *	0,00	0 (0,2)	(0,01 to 0,75)	0 (0,4)	(-0,25 to 0,368)	-0,31	0,22	1,637 (1,1)	(-1,1 to 1,637)	0,47 (1,82)	(-0,65 to -2,33)	-0,39	0,07	1,36(1,637)	(2,21 to -0,98)	1,22(2,4)	(0 to -0,9)	0,61	0,71
Type IV	Mid Tendon	Baseline	0 (0)	(0,04 to 0)	0,04 (0,7)	(0,346 to 0)	-0,07	0,18	0 (0,17)	(0,02 to 0,38)	0 (0,2)	(-0,09 to 0,22)	-0,26 *	0,01	0,581 (0)	(0,157 to -0,1)	0,12 (0)	(0,122 to -0,41)	-0,19	0,07	0,35(0,581)	(0,95 to 0,72)	0,19(0,86)	(0,24 to -2,15)	0,71	0,33
Type IV	Mid Tendon	4 months	0 (0,3)	(0,28 to 0,02)	-0,2 (0,6)	(-0,14 to -0,7)	0,19 *	0,00	0 (0,2)	(0,02 to 0,04)	0 (0,1)	(-0,23 to 0,077)	0,01	0,30	0,367 (0)	(0,613 to -0,76)	0,277 (1,22)	(0,454 to -0,56)	0,10	0,56	0,54(0,367)	(0,84 to 0,26)	0,82(0,14)	(-0,19 to -,14)	0,51	0,56
Type IV	Mid Tendon	8 months	0,05 (0,25)	(0,2 to 0,04)	-0,2 (0,3)	(-0,04 to -0,3)	0,28 *	0,00	0 (0,2)	(-0,13 to 0,08)	0 (0,2)	(-0,22 to 0,006)	-0,26	0,47	0,352 (0,35)	(-0,25 to 0,352)	0,282 (0,77)	(-0,1 to -0,98)	-0,70	0,12	0,31(0,352)	(0,51 to -0,75)	0(0,37)	(0 to -0,1)	0,33	0,78

Table S2: Ultrasound Tissue Characterization (UTC) descriptive data of echo-type (I-IV) at the proximal and mid-tendon at baseline, 4 and 8 months of training and competition porfessional female and male basketball players

n	Temp	Player Position	Jumping Leg	Sex	20_R_Type I	20_R_Type II	20_R_Type III	20_R_Type IV	20_L_Type I	20_L_Type II	20_L_Type III	20_L_Type IV	50_R_Type I	50_R_Type II	50_R_Type III	50_R_Type IV	50_L_Type I	50_L_Type II	50_L_Type III	50_L_Type IV
1	Baseline	Shooting Guard	R	F	45,6	53,4	0,8	0,2	68,9	30,9	0,2	0	65,7	34,2	0,1	0	77,2	22,4	0,3	0,1
2	Baseline	Shooting Guard	R	F	60,5	38,5	0,9	0,1	52,4	46,2	1	0,3	63	36,9	0,1	0	72,3	26,5	1	0,2
3	Baseline	Point Guard	L	F	54,9	40,4	3,5	1,2	70,2	29,3	0,4	0,1	78,8	21,2	0	0	74,8	25,2	0	0
4	Baseline	Pivot Wing	R	F	49,1	50,9	0	0	69,5	30,5	0	0	70,5	29,4	0	0	65,9	34,8	0	0
5	Baseline	Center	R	F	66,2	33,8	0	0	90,9	9,1	0	0	66,4	33,6	0	0	88,6	11,4	0	0
6	Baseline	Shooting Guard	R	F	54,4	44	1,2	0,3	55,5	42,4	0,7	0,3	69,7	29,1	0,9	0,3	4,2	34,6	1,1	0,2
7	Baseline	Center	L	F					56,2	43,1	0,6	0,1					50,8	48,9	0,3	0
8	Baseline	Pivot Wing	L	F	60	39,6	0,3	0,1	56,8	42,8	0,3	0,1	52,9	45,8	1,1	0,2	52,8	45,6	1,3	0,3
9	Baseline	Center	R	F	50,8	48	1	0,2	43,2	56,2	0,5	0,1	66,6	31,4	1,5	0,5	70,7	29,1	0,2	0
10	Baseline	Small Forwards	L	F	60,4	37,9	1,3	0,4	57,6	42,2	0,2	0	61,1	36,3	1,8	0,8	61,8	33,3	3,4	1,5
11	Baseline	Small Forwards	R	F	36,3	62,3	1,2	0,3	62,4	35,9	1,4	0,3	63,7	35,8	0,4	0,2	70,3	27,7	1,5	0,6
12	Baseline	Pivot Wing	R	F	51,1	47,1	1,5	0,3	51,8	45,8	1,9	0,5	55,2	44,2	0,5	0,1	53,9	43,6	2,1	0,5
13	Baseline	Small Forwards	L	F	58,1	36,3	3,9	1,7	52	47,2	0,6	0,1	48,6	45,9	4,1	1,4	66,8	32,5	0,6	0,1
14	Baseline	Point Guard	R	F	68,7	30,7	0,4	0,1	50,6	49	0,2	0,1	60,7	39,1	0,3	0	55,2	44,3	0,3	0,1
15	Baseline	Point Guard	R	F	51,8	47,8	0,4	0	50,1	47,4	2	0,4	42,7	56,9	0,4	0	59,2	40,3	0,5	0
16	Baseline	Small Forwards	R	F	65,2	34,8	0	0	63,7	36,2	0,1	0	66,8	32,1	0,9	0,2	72,1	27,8	0	0
17	Baseline	Point Guard	R	F					60,3	36,9	2,1	0,7					70,8	28,6	0,4	0,1
18	Baseline	Point Guard	R	F	64,1	35,9	0	0	69,8	30,2	0	0	73,2	26,7	0,1	0	71,1	28,9	0	0
19	Baseline	Small Forwards	R	F	36,2	63,5	0,2	0	58,8	38,9	1,7	0,7	47,5	51,5	0,9	0,2	66,4	31,4	1,6	0,5
20	Baseline	Center	L	F	54,7	42,7	2	0,6	49,2	48,8	1,7	0,3	56,7	41,6	1,2	0,5	58,7	40,9	0,4	0,1
21	Baseline	Center	R	F	57,9	38,5	2,8	0,8	68	30,1	1,5	0,5	78,1	21,4	0,3	0,2	78,9	18,2	1,8	1,1
22	Baseline	Point Guard	R	F	53,2	46,4	0,3	0	45,6	51,5	2,3	0,6	69,4	29,7	0,7	0,2	65,9	32,5	1,1	0,5
23	Baseline	Small Forwards	R	F	48,8	47,1	3,2	0,9					53,1	46,2	0,7	0,1				
24	Baseline	Center	R	M	55	45	0	0	76,4	23,6	0	0	62,3	37,7	0	0	77,4	22,6	0	0
25	Baseline	Small Forwards	R	M	77,3	22,7	0	0	81,3	18,7	0	0	85	15	0	0	62,5	37,5	0	0
26	Baseline	Small Forwards	R	M	61,1	38,9	0	0	73,9	26,1	0	0	69,8	30,2	0	0	71,5	28,5	0	0
27	Baseline	Center	L	M	65,1	34,8	0,1	0	46,5	50,2	2,9	0,3	76,2	23,3	0,4	0,2	83,2	16,8	0	0
28	Baseline	Point Guard	R	M	59,5	39,8	0,6	0,2	65,3	34,7	0	0	63,4	36,6	0	0	73,4	26,6	0	0
29	Baseline	Shooting Guard	R	M					65,8	34,2	0	0					78,4	21,6	0	0
30	Baseline	Small Forwards	R	M	72,3	27,7	0	0	63,6	36,4	0	0	61,9	38,1	0	0	50,9	49,1	0	0
31	Baseline	Small Forwards	R	M	80,8	19,2	0	0	64	36	0	0	83,1	16,9	0	0	76,7	23,2	0	0
32	Baseline	Pivot Wing	R	M	57,9	42,1	0	0	63,6	36,4	0	0	61,3	38,7	0	0	71,2	28,8	0	0
33	Baseline	Shooting Guard	L	M	55,7	44,3	0	0	70,3	29,7	0	0	77,1	22,9	0	0	68	32	0	0
34	Baseline	Shooting Guard	R	M	72,5	27,5	0	0	70,8	29,2	0	0	77	23	0	0	82,8	17,2	0	0
35	Baseline	Shooting Guard	R	M	66,7	33,3	0	0	75,7	24,9	0	0	73,4	26,6	0	0	53,5	46,5	0	0
36	Baseline	Center	R	M	71,3	28,7	0	0	68,3	31,7	0	0	75,5	24,4	0,1	0	70,5	29,5	0	0
37	Baseline	Pivot Wing	R	M	47,9	52,1	0	0	41,3	58,7	0	0	63	37	0	0	73,7	43,6	0	0
38	Baseline	Center	R	M	72	28	0	0	74,3	25,7	0	0	71,7	28,2	0	0	73,5	26,5	0	0
39	Baseline	Shooting Guard	R	M	54,7	44,1	1	0,2	86,3	13,5	0,1	0,1	81,8	18,2	0	0	82,1	17,9	0	0
40	Baseline	Small Forwards	R	M	49,7	50,3	0	0	45,7	53,9	0,4	0,1	68,8	31,2	0	0	80,3	19,7	0	0
41	Baseline	Pivot Wing	L	M					64,2	35,8	0	0					48,6	51,4	0	0
42	Baseline	Pivot Wing	R	M	75,5	24,5	0	0	76	24	0	0	82,1	17,9	0	0	65,3	34,7	0	0
43	Baseline	Shooting Guard	R	M	62,5	33,6	2,4	1,5	41,7	44,2	10	4,1	71,4	28,5	0,1	0	73,5	26,5	0	0
44	Baseline	Point Guard	L	M	65	34,8	0,1	0	84,7	15,3	0	0	79	21	0	0	87	13	0	0
1	4 Months	Shooting Guard	R	F	43,9	45,8	8,4	1,9	63,4	34,9	1,3	0,5	42,7	47,5	7,3	2,6	62,6	33,2	2,5	1,7
2	4 Months	Shooting Guard	R	F	46,8	36,7	11,4	5,1	59,7	37,4	2	0,9	63,7	31,1	3,8	1,4	69,2	28,8	1,3	0,7
3	4 Months	Point Guard	L	F	56,5	40,7	2	0,8	70,6	27,6	1,1	0,7	63,1	33,5	2,2	1,1	70,5	23,2	4,1	2,3
4	4 Months	Pivot Wing	R	F	63,3	34,9	1,3	0,5	45,6	52,2	1,7	0,5	59,6	38,2	1,6	0,6	64,5	33,4	1,5	0,7
5	4 Months	Center	R	F	59,3	36,9	2,3	1,4	49,3	50,2	0,4	0,1	60,2	38,5	1	0,3	67	32,4	0,5	0,2
6	4 Months	Shooting Guard	R	F	64,4	34,1	1	0,4	54,3	40,9	3,1	1,8	67	29,8	1,9	1,3	64,2	33,6	1,6	0,6
7	4 Months	Center	L	F	48,5	51,3	0,2	0	44,5	54,2	1	0,3	48,7	51,2	0,2	0	47,5	51,8	0,6	0,1
8	4 Months	Pivot Wing	L	F	49,2	44,4	4,3	2	41,8	46,5	8,3	3,4	43,7	53	2,7	0,7	53,2	43,8	2,2	0,7
9	4 Months	Center	R	F	63,6	27,9	4,7	3,8	51,2	45,8	2,2	0,8	60,6	36,2	2,2	1	61,5	36,9	1,3	0,3
10	4 Months	Small Forwards	L	F	62	34,2	2,6	1,3	52,7	45,4	1,5	0,3	57,2	36,7	3,8	2,4	56	40,5	2,4	1
11	4 Months	Small Forwards	R	F	64,8	34,8	0,3	0,2	56,8	43	0,1	0	58,9	40,4	0,5	0,2	69,4	30,5	0	0
12	4 Months	Pivot Wing	R	F	62,6	37	0,3	0,1	51,6	43,7	3,2	1,6	71,1	28,3	0,4	0,2	55,4	43,2	1,2	0,2
13	4 Months	Small Forwards	L	F	68,5	30,6	0,5	0,4	64,6	32,6	1,5	1,2	65,9	33,8	0,2	0,1	73,8	25,7	0,3	0,2
14	4 Months	Point Guard	R	F	64,3	35,2	0,4	0,1	62,3	37,3	0,3	0,1	73,6	26,3	0,1	0	65,1	34,7	0,2	0,1
15	4 Months	Point Guard	R	F	67	32,4	0,4	0,1	75,6	24,2	0,2	0,1	60,1	38,8	0,9	0,2	76,4	23,4	0,2	0
16	4 Months	Small Forwards	R	F	60,1	39,9	0	0	76,1	23,9	0	0	74,8	25,2	0	0	70,1	29,8	0	0
17	4 Months	Point Guard	R	F	70,5	29,4	0,1	0	73,4	26,3	0,2	0,1	78,6	21,3	0,1	0	84,8	15,1	0	0

18	4 Months	Point Guard	R	F	67,2	32,4	0,2	0,1	65,2	34,2	0,3	0,3	83,2	16,8	0	0	79,2	20,8	0	0
19	4 Months	Small Forwards	R	F	73,4	24,8	1,1	0,6	72,2	26,9	0,5	0,3	77	23	0	0	81,8	17,8	0,3	0,2
20	4 Months	Center	L	F	75,2	24,7	0,1	0,1	67,5	32	0,3	0,2	79,9	19,8	0,1	0,1	80,9	18,2	0,4	0,4
21	4 Months	Center	R	F	65	34,8	0,1	0	80,3	19,7	0	0	85	14,8	0,1	0,1	81,2	18,7	0,1	0
22	4 Months	Point Guard	R	F	60	40	0	0	66	33,9	0	0	71,3	28,7	0	0	82,6	17,4	0	0
23	4 Months	Small Forwards	R	F	61,5	38,3	0,2	0	63,4	36,6	0,1	0	69,2	30,6	0,1	0	68,3	31,3	0,2	0,2
24	4 Months	Center	R	M	64,5	35,5	0	0	67,2	32	0	0	77	23	0	0	70,4	29,6	0	0
25	4 Months	Small Forwards	R	M					54,2	45,8	0	0					87,2	12,8	0	0
26	4 Months	Small Forwards	R	M	70,7	29,2	0	0	76,3	23,7	0	0	86,1	13,9	0	0	84,4	15,6	0	0
27	4 Months	Center	L	M	75,7	23,9	0,3	0,1	67	32,1	0,7	0,2	81,7	18,3	0	0	77,9	22,1	0	0
28	4 Months	Point Guard	R	M	66,7	29,9	2,2	1,2	68,5	31,5	0	0	69,5	30,4	0,1	0	68	31,9	0	0
29	4 Months	Shooting Guard	R	M	74,2	25,8	0	0	73,7	26,3	0	0	62,2	37,8	0	0	78,3	21,7	0	0
30	4 Months	Small Forwards	R	M	18,1	75,5	6	0,4	21,3	75,4	3,1	0,2	44,8	55,2	0	0	41,7	58,3	0	0
31	4 Months	Small Forwards	R	M	88,6	11,4	0	0	63,2	36,4	0,3	0,1	91,9	8,1	0	0	76,1	23,8	0,1	0
32	4 Months	Pivot Wing	R	M	41,6	58,4	0	0					69	31	0	0				
33	4 Months	Shooting Guard	L	M	33,4	66,6	0	0	26,7	73,3	0	0	92,1	7,9	0	0	46	54	0	0
34	4 Months	Shooting Guard	R	M	62,3	37,6	0,1	0,1	61,6	37,4	0,8	0,2	69,9	29,9	0,1	0,1	68,5	31,5	0	0
35	4 Months	Shooting Guard	R	M	79,5	20,5	0	0	54	46	0	0	40,5	59,5	0	0	79,6	20,4	0	0
36	4 Months	Center	R	M	68,6	31,3	0,1	0	63,6	29,9	4,1	2,4	74,8	24,8	0,3	0,1	65,7	33,5	0,6	0,2
37	4 Months	Pivot Wing	R	M	68,4	31,6	0	0	31,7	68,3	0	0	66,4	33,6	0	0	62,9	37,1	0	0
38	4 Months	Center	R	M	55,1	44,5	0,3	0,1	69,1	30,9	0	0	47,5	52,5	0,1	0	60,9	39,1	0	0
39	4 Months	Shooting Guard	R	M	77	21,5	1	0,5	72,3	26,7	0,6	0,4	68,5	30,9	0,4	0,2	71,2	28,4	0,3	0,1
40	4 Months	Small Forwards	R	M	20,2	59,7	16,5	3,6	35,8	61,7	2,1	0,4	51,5	48,5	0	0	62	38	0	0
41	4 Months	Pivot Wing	L	M	64,4	35,6	0	0	57,8	42,2	0	0	31,9	68,1	0	0	29,7	70,3	0	0
42	4 Months	Pivot Wing	R	M	75	24,9	0	0	67,9	32	0	0	68,5	31,5	0	0	66,4	33,6	0	0
43	4 Months	Shooting Guard	R	M	72,9	24,7	1,7	0,8	70	21,2	4,5	4,3	75,3	24,3	0,3	0,1	74,4	25,1	0,3	0,2
44	4 Months	Point Guard	L	M	70,4	25	2,3	2,3	75,9	24,1	0	0	59,8	40,1	0	0	72,7	27,3	0	0
1	8 Months	Shooting Guard	R	F	68,1	31,8	0,1	0	79,9	19,7	0,2	0,2	72,7	27,3	0,1	0	86,6	13,3	0,1	0
2	8 Months	Shooting Guard	R	F	70,2	29,8	0,1	0	74,6	25,1	0,2	0	73,3	26,4	0,1	0,1	84	15,9	0	0
3	8 Months	Point Guard	L	F	66,5	33,4	0,1	0	79,1	20,8	0,1	0	84,7	15,2	0,1	0	83,7	16,2	0	0
4	8 Months	Pivot Wing	R	F	58,4	41,6	0	0	70,5	29,5	0	0	77,1	22,9	0	0	78,8	21,2	0	0
5	8 Months	Center	R	F	66,9	33,1	0	0	72,7	27,2	0,1	0	74	26	0	0	79,2	20,8	0	0
6	8 Months	Shooting Guard	R	F	63,7	36,2	0,1	0	71,9	28,1	0	0	82,7	17,3	0	0	74	25,1	0,6	0,2
7	8 Months	Center	L	F	65	35	0	0	75,8	24,2	0	0	70,6	29,4	0	0	76,2	23,7	0	0
8	8 Months	Pivot Wing	L	F	65,6	34,4	0,1	0	74,7	23,8	0,9	0,6	78,2	21,7	0	0	80,3	19,7	0	0
9	8 Months	Center	R	F	72,2	27	0,6	0,2	70	29,2	0,6	0,2	81,4	18,4	0,1	0	79,4	20,6	0	0
10	8 Months	Small Forwards	L	F	62,7	35,3	1,1	0,9	72,7	26,6	0,4	0,2	67,1	32,5	0,2	0,2	80,5	19,5	0	0
11	8 Months	Small Forwards	R	F	78,7	21,3	0	0	81	18,9	0	0	77,6	22,4	0	0	77,9	21,9	0,2	0,1
12	8 Months	Pivot Wing	R	F	61,7	38,1	0,1	0,1	78,5	19,7	1,2	0,6	72,7	27,3	0	0	81,5	18,5	0	0
13	8 Months	Small Forwards	L	F	67	32,9	0	0	68	32	0	0	80,3	19,7	0	0	79,3	20,7	0	0
14	8 Months	Point Guard	R	F	68,8	30,7	0,4	0,1	77,8	22	0,2	0	83,5	16,4	0	0	80,1	19,9	0	0
15	8 Months	Point Guard	R	F	61,3	38,4	0,3	0,1	66,2	33,8	0	0	72,1	27,9	0	0	77	23	0	0
16	8 Months	Small Forwards	R	F	74,3	25,7	0	0					76,4	23,6	0	0				
17	8 Months	Point Guard	R	F	82,2	17,6	0,1	0	65,7	34,3	0	0	76,3	23,6	0,1	0	82,5	17,5	0	0
18	8 Months	Point Guard	R	F	68,7	31,3	0	0	79,8	20,2	0	0	83,5	16,5	0	0	87,6	12,4	0	0
19	8 Months	Small Forwards	R	F	81,3	184	0,2	0,1	71,3	28,4	0,2	0,1	84,4	15,5	0	0	76,3	23,7	0	0
20	8 Months	Center	L	F	57,5	42,2	0,2	0	52,5	46,7	0,7	0,1	80,1	19,9	0	0	84,9	15,1	0	0
21	8 Months	Center	R	F	70,8	28	1	0,3	73	25,7	1	0,4	89,3	10	0,3	0,5	91,3	8,3	0,2	0,2
22	8 Months	Point Guard	R	F	66,5	33,3	0,1	0,1	79,3	20,7	0	0	84,4	15,6	0	0	81,8	17,8	0,3	0,1
23	8 Months	Small Forwards	R	F	70,7	29,3	0	0	73,1	25	1,3	0,6	72,6	27,4	0	0	81,7	18,1	0,1	0,1
24	8 Months	Center	R	M	65,3	34,7	0	0	72,8	27,2	0	0	78,6	21,4	0	0	70,3	29,7	0	0
25	8 Months	Small Forwards	R	M	73,6	26,4	0	0	78,3	21,7	0	0	83,7	16,3	0	0	81,6	18,4	0	0
26	8 Months	Small Forwards	R	M	62,,9	37,1	0	0	69,4	30,6	0	0	85,7	14,3	0	0	84	16	0	0
27	8 Months	Center	L	M	74,5	22,5	2,1	1	54,7	32,2	9,3	3,7	61,3	38,4	0,3	0	62,3	34,2	2,9	0,6
28	8 Months	Point Guard	R	M	41,1	41,1	13,1	4,6	39,9	57,3	2,5	0,3	49,7	48,7	1,4	0,2	47,1	49,8	2,6	0,5
29	8 Months	Shooting Guard	R	M					61	39	0	0					77,4	22,6	0	0
30	8 Months	Small Forwards	R	M	83,5	16,5	0	0	63,8	35,4	0,6	0,2	78,2	20,5	0,7	0,6	72	28	0	0
31	8 Months	Small Forwards	R	M	51,6	48,2	0,1	0	43,9	51,2	4,1	0,8	62,9	36,9	0,2	0	50	45,9	3,4	0,7
32	8 Months	Pivot Wing	R	M	63,2	36,7	0	0	55,4	44,5	0,1	0	77,5	22,5	0	0	71,9	28,1	0	0
33	8 Months	Shooting Guard	L	M	64,6	35,4	0	0	55,2	44,8	0	0	68,9	31	0	0	67,1	32,9	0	0
34	8 Months	Shooting Guard	R	M	66,2	33,8	0	0	54,1	44,5	1,3	0,2	71,2	28,4	0,3	0,1	65,3	33,9	0,7	0,2
35	8 Months	Shooting Guard	R	M	70,3	29,1	0,4	0,2	75,7	24,1	0,1	0	73,9	26,1	0	0	83,5	16,5	0	0
36	8 Months	Center	R	M	42,3	55,4	2	0,3					49,2	50,6	0,2	0				

37	8 Months	Pivot Wing	R	M	48,7	51	0,3	0	57,9	42	0,1	0	40,3	56	3,1	0,6				
38	8 Months	Center	R	M	66	33,2	0,7	0,2	46,7	53,1	0,2	0	67,6	32,1	0,2	0,1	55,3	33,7	0,1	0
39	8 Months	Shooting Guard	R	M	56,8	39,4	2,6	1,2	66,7	32,6	0,5	0,2	77,4	22	0,4	0,2	53,6	45,9	0,4	0,1
40	8 Months	Small Forwards	R	M	56,9	37,5	4	1,6	57,8	35,8	4,6	1,8	70	30	0	0	69,7	30,2	0	0
41	8 Months	Pivot Wing	L	M	83,3	16,7	0	0	68,2	31,8	0	0	86,5	13,5	0	0	75,3	24,7	0	0
42	8 Months	Pivot Wing	R	M	61,5	38,4	0,1	0	55,9	43,9	0,1	0	68,3	31,6	0,1	0	65,8	33,6	0,5	0,1
43	8 Months	Shooting Guard	R	M	62,8	29,9	4,6	2,7	54,9	25,3	10,8	9,1	73,3	26,3	0,3	0,1	60,8	35,1	2,8	1,2
44	8 Months	Point Guard	L	M	79,3	20,7	0	0	54,9	34,8	6,1	4,2	76,9	23,1	0	0	65,9	33,7	0,3	0,1
